# Supplementary material for: Immunomagnetic sequential ultrafiltration (iSUF) platform for enrichment and purification of extracellular vesicles from biofluids
Source: Sci Rep. 2021 Apr 13;11:8034. doi: 10.1038/s41598-021-86910-y (PMC8044115; doi:10.1038/s41598-021-86910-y)
Supplement: Supplementary file 1 — Supplementary Information. [file 41598_2021_86910_MOESM1_ESM.docx]

**Immunomagnetic Sequential Ultrafiltration (iSUF) Platform for Enrichment and Purification of Extracellular Vesicles from Biofluids**

**Jingjing Zhang,^1^ Luong TH Nguyen,^1^ Richard Hickey,^1^ Nicole Walters,^1^ Xinyu Wang,^1^ Kwang Joo Kwak,^1^ L. James Lee, ^1^ Andre F. Palmer,^1^ Eduardo Reátegui^1,2*^**

^1^ William G. Lowrie Department of Chemical and Biomolecular Engineering, The Ohio State University, Columbus OH 43210

^2^ Comprehensive Cancer Center, The Ohio State University, Columbus, OH 43210

*corresponding author: [reategui.8@osu.edu](mailto:reategui.8@osu.edu)

**Supplementary Information**

**Atomic force microscopy (AFM)**

A clean mica substrate was vapor-phase coated with 3-aminopropyltriethoxysilane (APTES, Millipore Sigma, Burlington, MA) in a vacuum chamber and then dried overnight at 65 °C. Subsequently, 10 µL of purified EVs were incubated on the surface for 30 min at RT. Unbound EVs were extensively rinsed with PBS and then with DI water. The samples were air-dried again before imaging using an AFM (Asylum Research MFP-3D-BIO AFM, Oxford Instruments, Abingdon, United Kingdom).

**Scanning electron microscopy (SEM)**

Clean coverslips were soaked in 0.25 mg/mL Zetag solution (BASF, Southfield, MI) for 30 min, followed by overnight air drying at RT. Purified EVs were attached to the coated coverslip for 30 min at RT by physisorption. EVs were fixed in 2% glutaraldehyde (MilliporeSigma, Burlington, MA) and 0.1 M sodium cacodylate solution (Electron Microscopy Sciences, Hatfield, PA) for 3h. After washing with 0.1 M sodium cacodylate solution, EVs were incubated in 1% osmium tetraoxide (Electron Microscopy Sciences) and 0.1 M sodium cacodylate for 2h. The sample was subsequently rinsed with 0.1 M sodium cacodylate solution before dehydration in increasing concentrations of ethanol (50, 70, 85, 95, and 100%, ThermoFisher Scientific) for 30 min each. Next, the samples were transferred to a CO_2_ critical point dryer (Tousimis, Rockville, MD). Finally, the samples were coated with ~ 2 nm of gold using a sputtering machine (Leica EM ACE 600, Buffalo Grove, IL) and imaged using SEM (Apreo ii, FEI, Thermo Fisher Scientific).

**Transmission electron microscopy (TEM)**

3 μL EVs purified from CCM using iSUF were applied to a glow discharged lacey carbon-coated copper grid (400 mesh, Pacific Grid-Tech, San Francisco, CA) and flash-frozen in liquid ethane using an automated vitrification device (FEI Vitrobot Mark IV, FEI, Hillsboro, OR). The sample was then visualized in a Glacios Cryo-TEM (Thermo Fisher Scientific).

**Protein extraction and quantification**

EV samples were lysed in radioimmunoprecipitation assay (RIPA) buffer (Abcam, Cambridge, MA) with the addition of Thermo Scientific Halt Protease and Phosphatase Inhibitor Cocktails for 15 min on ice. EV samples (with/without lysis) were then pipetted to a 96-well plate, and their protein concentrations were quantified using a Pierce Rapid Gold BCA Protein Assay kit (Thermo Fisher Scientific). EV protein concentration was determined by subtracting the amount of free protein (without lysis) from the total (with lysis) in the purified EV sample.

**Sodium dodecyl sulfate-polyacrylamide gel electrophoresis (SDS-PAGE)**

Proteins in the final product were denatured and reduced in the presence of NuPAGE Reducing Agent in NuPAGE LDS Sample Buffer at 95 ºC for 10 min. The proteins were then separated in a mini gel tank (ThermoFisher Scientific) using NuPAGE 4-12% Bis-Tris Protein Gel in NuPAGE MOPS SDS Running Buffer for 50 min at 200 V. After separation, the proteins were stained with Coomassie Brilliant Blue G-250 Dye.

**Western blotting**

After separation by SDS-PAGE, proteins were transferred onto a polyvinylidene fluoride (PVDF) membrane (Thermo Fisher Scientific) and then blocked with 3% bovine serum albumin (BSA) and 0.05% Tween in PBS for 1 hr at RT. Primary antibodies against tetraspanin surface markers such as CD63 and CD9 were incubated with the EVs overnight at 4 °C (Santa Cruz Biotechnology, Inc Dallas, TX). The next day, the PVDF membrane was incubated with an HRP conjugated secondary antibody for 1h at RT. Finally, the sample was incubated with SuperSignal West Femto Maximum Sensitivity Substrate for 5 min at RT before imaging using a C-digit blot scanner (LI-COR, Lincoln, NE).

**RNA quantification**

Total RNA was extracted with QIAzol Lysis Reagent and then purified using a miRNeasy Mini kit according to the manufacturer’s protocol (Qiagen, Germantown, MD). After purification, the RNA concentration was quantified using a Qubit microRNA Assay Kit at excitation/emission wavelengths of 500/525 nm.


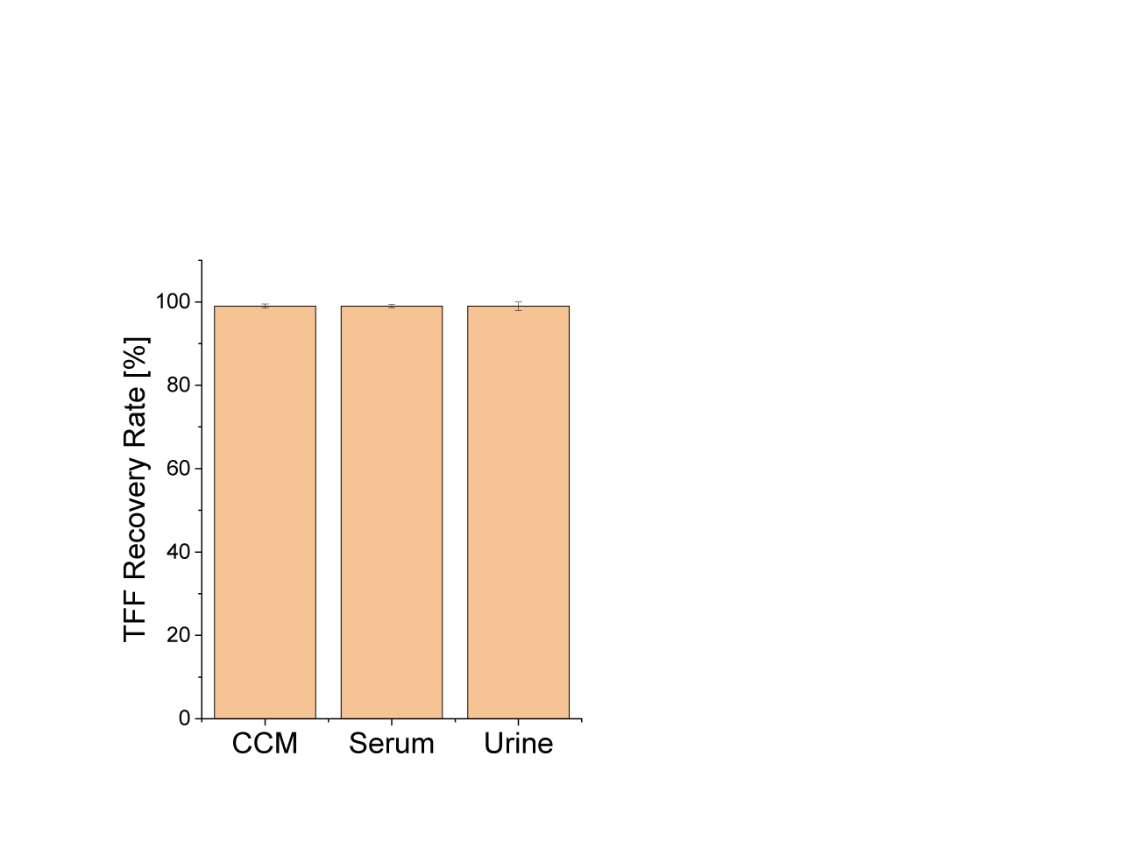


**Supplementary Figure 1.** EV recovery rate after TFF processing. Cell culture medium (n = 5), serum (n = 5), and urine (n = 5) were purified using TFF and enriched into a volume of 2 mL.


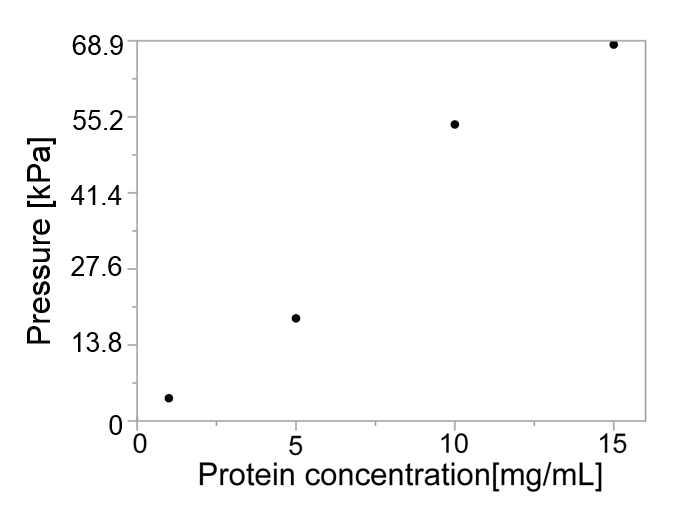


**Supplementary Figure 2.** Pressure in the TFF stage of iSUF when processing bovine serum albumin (BSA) solutions at different concentrations using a fixed flow rate of 35 mL/min. 15 mg/mL was the maximum protein concentration in the TFF system to maintain the system pressure below 68.9 kPa.

**
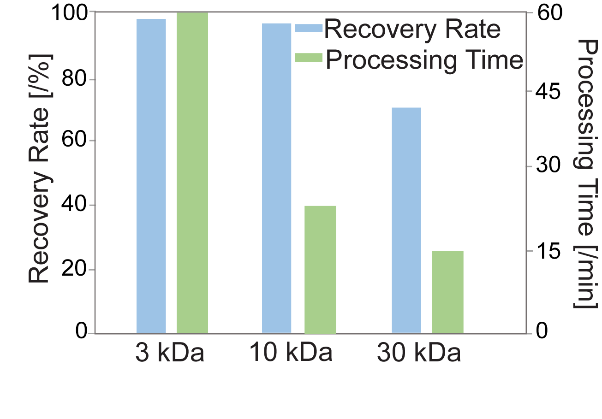
**

**Supplementary Figure 3.** EV recovery rate and processing time of centrifugal units (iSUF, stage 2) with different MWCOs. 3 kDa, 10 kDa, and 30 kDa MWCO centrifugal units obtained over 99%, 95%, and 70% recovery rate, respectively. They took 60 min (3 kDa MWCO), 20 min (10 kDa MWCO), and 15 min (30 kDa MWCO) to spin down the sample to a final volume of 100 µL.

**
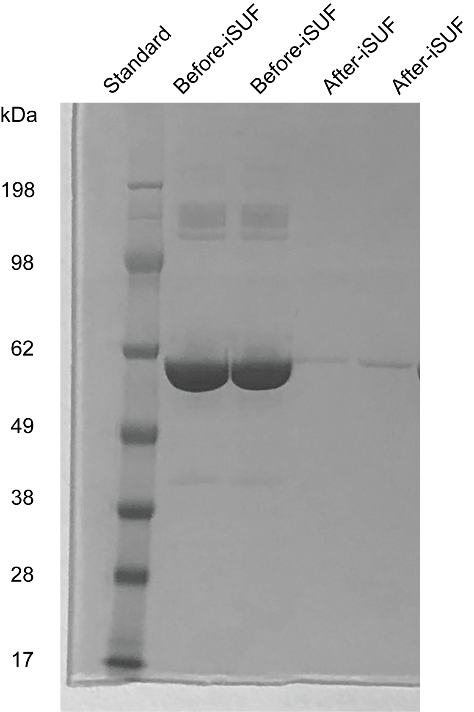
**

**Supplementary Figure 4.** SDS-PAGE of 10% BSA solution before and after iSUF processing. BSA was extensively removed after iSUF processing.

**
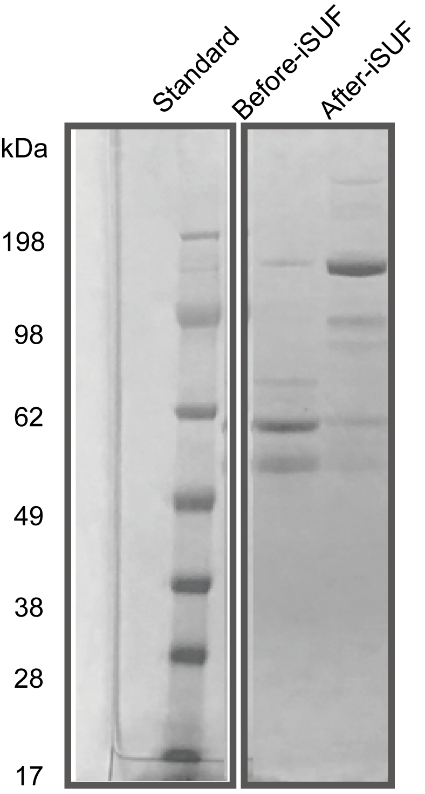
**

**Supplementary Figure 5.** SDS-PAGE of CCM before and after iSUF processing. BSA was extensively removed after iSUF processing.

**
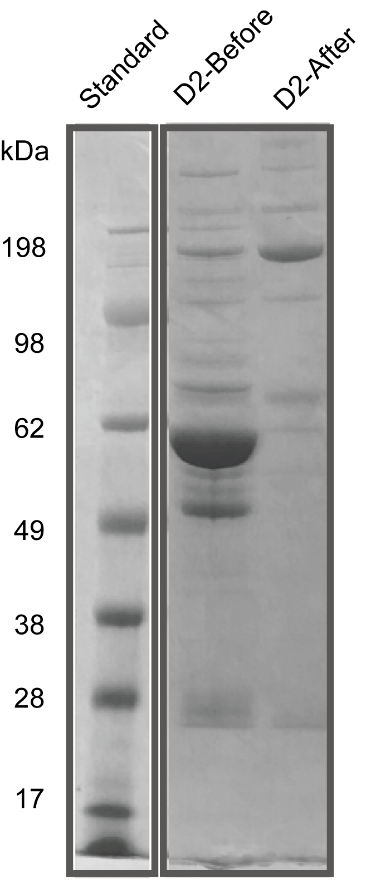
**

**Supplementary Figure 6.** SDS-PAGE of serum samples before and after iSUF processing. HSA was extensively removed after iSUF processing.

**
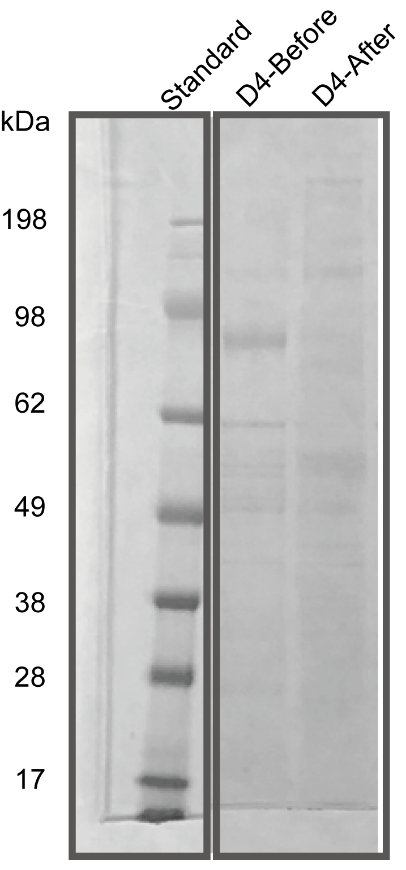
**

**Supplementary Figure 7.** SDS-PAGE of urine samples before and after iSUF processing. HSA and TPH were extensively removed after iSUF processing.

**
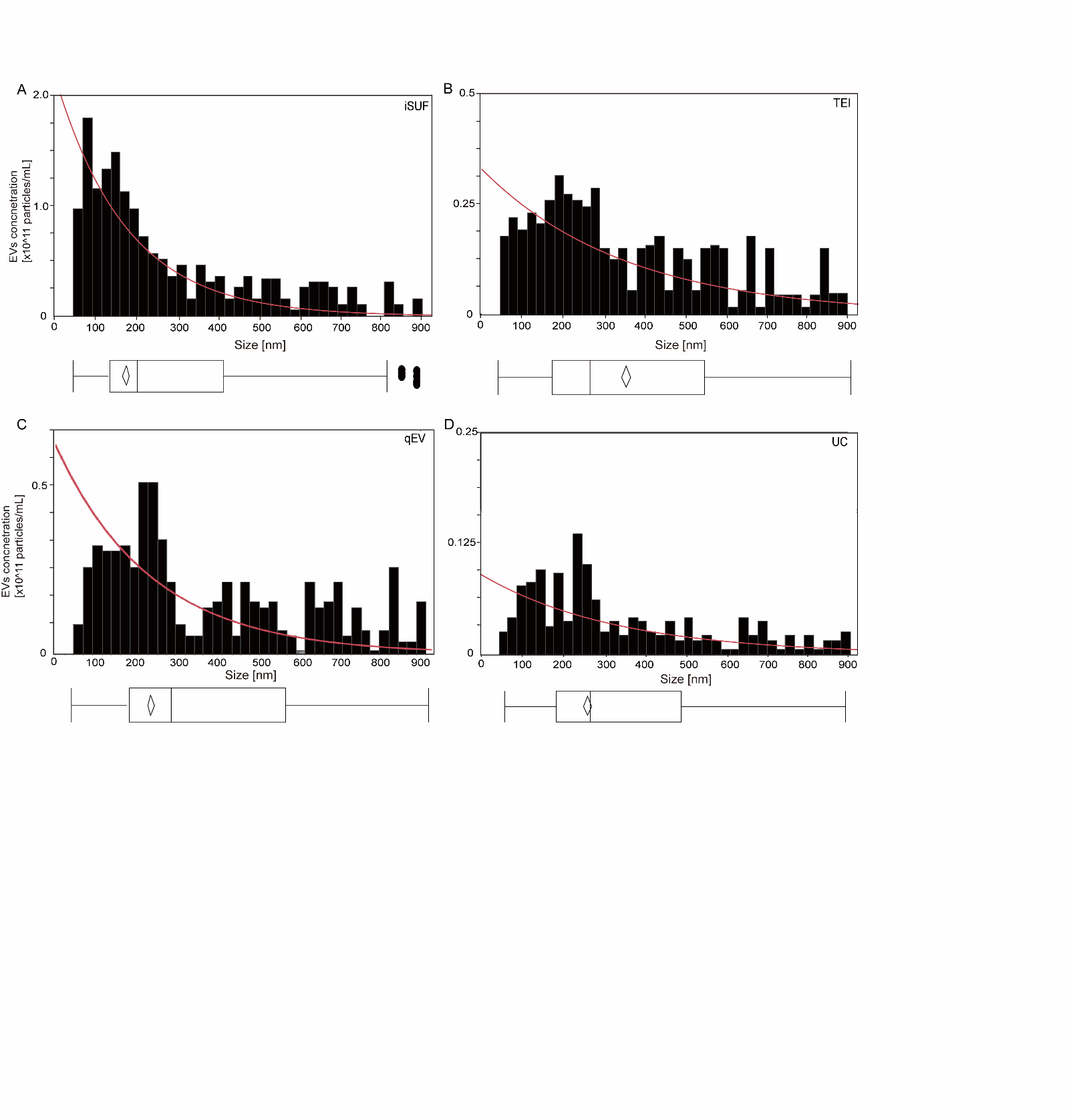
**

**Supplementary Figure 8.** The separated size distribution of serum-EVs isolated using iSUF (A), TEI (B), qEV (C), and UC (D). They presented a right-skewed distribution. Red lines were the exponential fitting generated in the JMP.

**
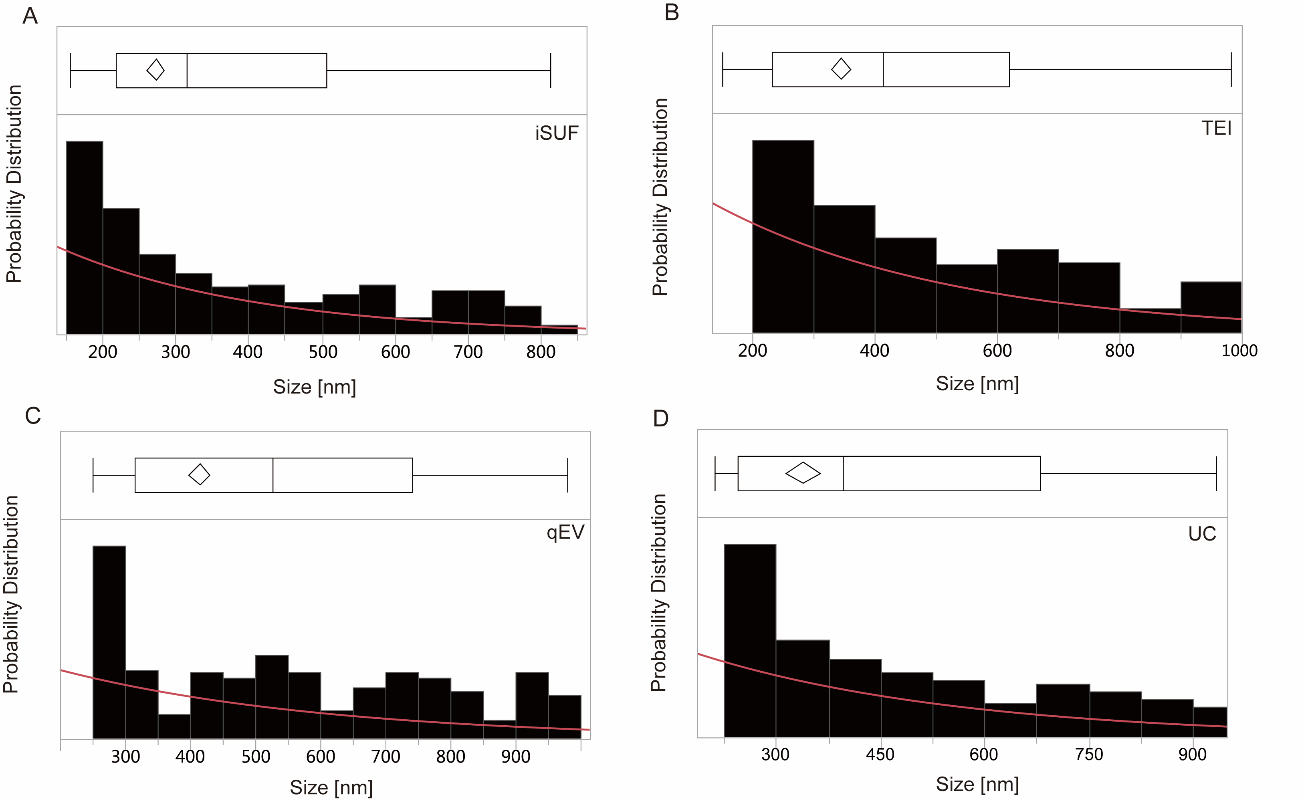
­­**

**Supplementary Figure 9.** The probability distribution of serum-EVs isolated using iSUF (A), TEI (B), qEV (C), and UC (D). The exponential distribution of these plots was validated using Kolmogorov D fitting test (p > 0.05).


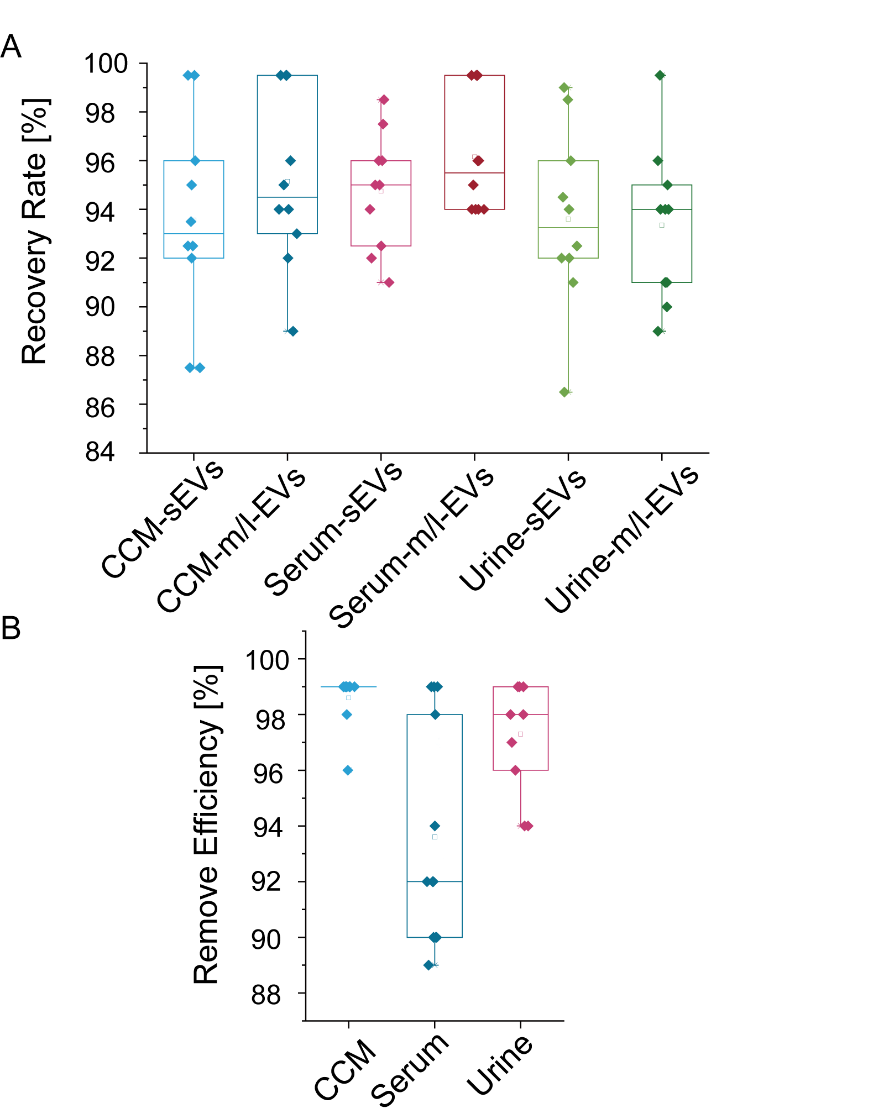


**Supplementary Figure 10. A).** Repeatability evaluation of EV recovery rate for samples processed by iSUF. Cell culture medium (n = 10), serum (n = 10), and urine (n =10) were purified by iSUF and enriched into a final volume of 100 µL. **B).** Repeatability evaluation of protein removal efficiency for samples processed by iSUF.


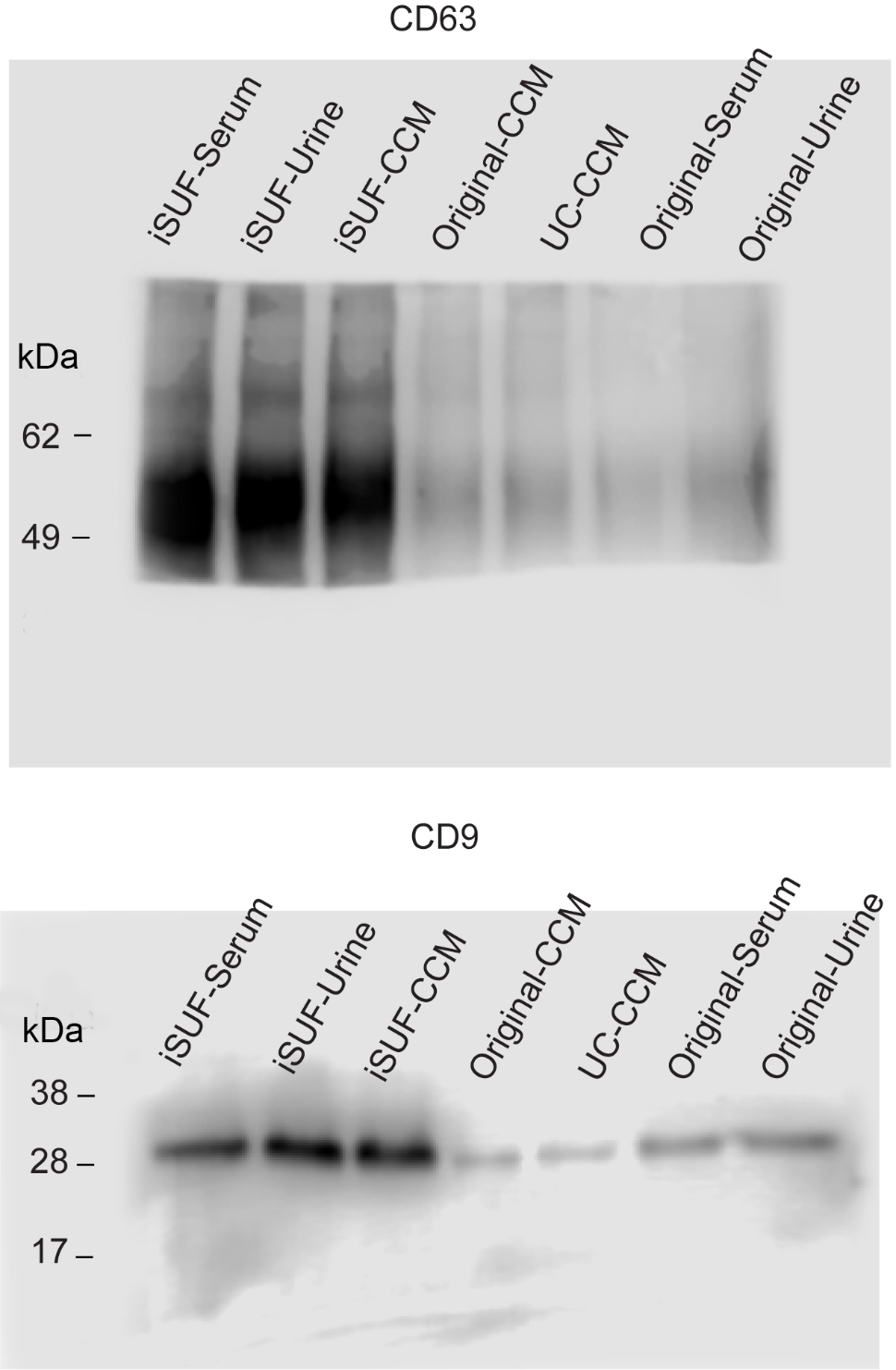


**Supplementary Figure 11.** Full-length western blots for cropped images in Fig. 4E.


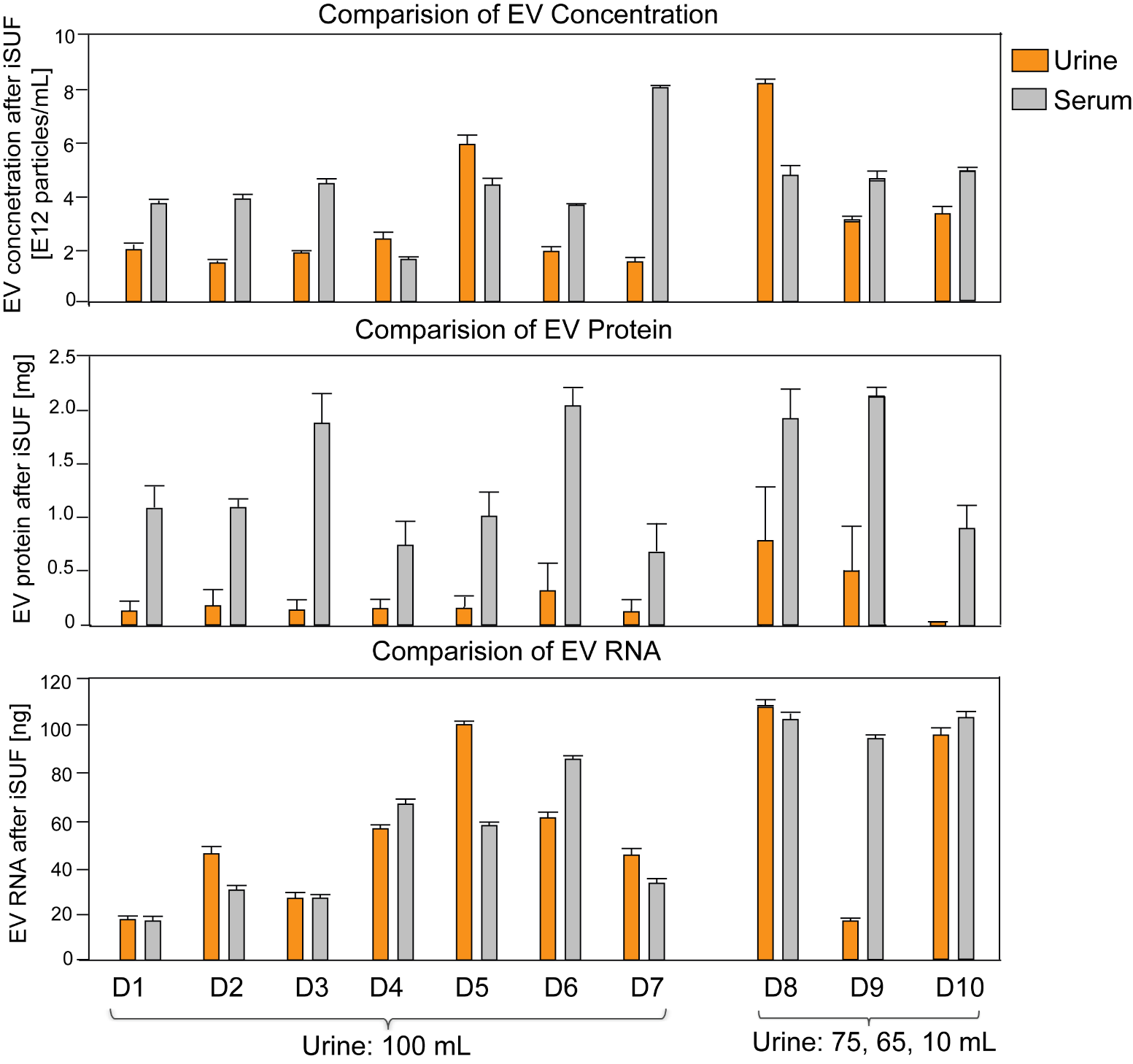


**Supplementary Figure 12.** Comparison of EV concentration, protein, and RNA content in iSUF-urine and iSUF-serum samples from seven healthy donors. The EV concentration and RNA content showed comparable values between urine and serum (n = 10; p > 0.05), while protein content in urine was 10 times lower than in serum.

**
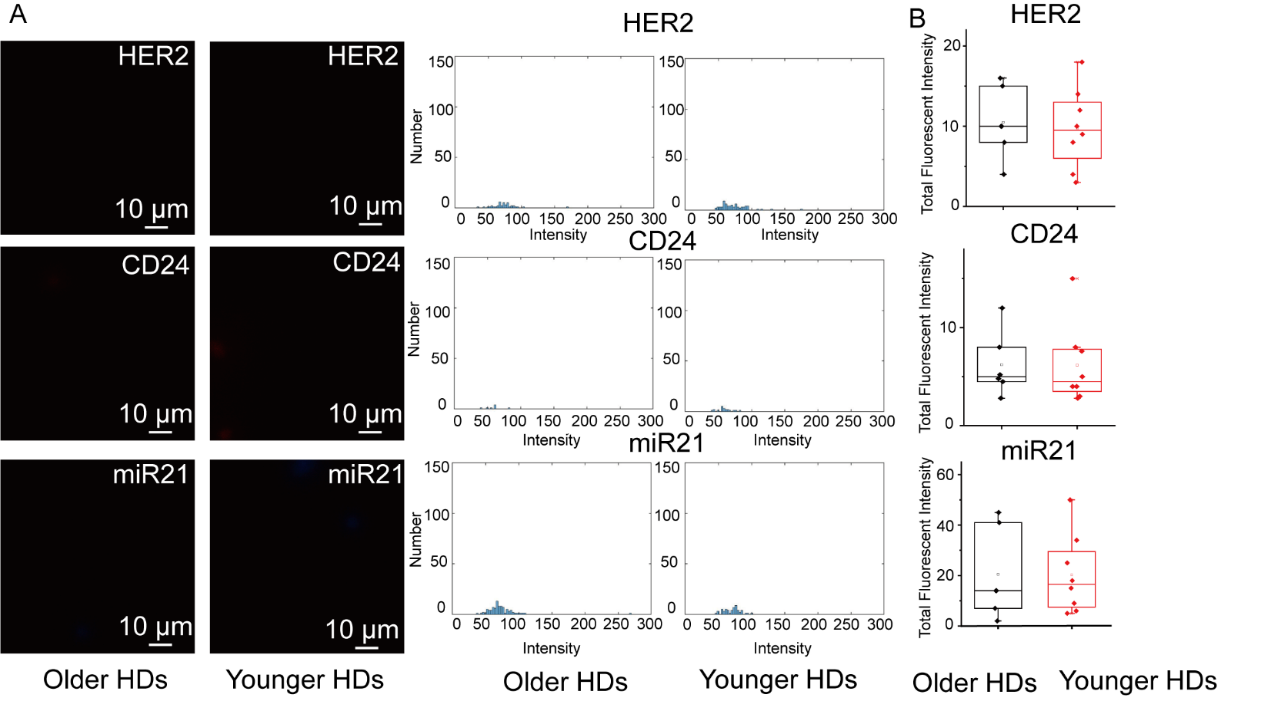
**

**Supplementary Figure 13.** Levels of expression of BC biomarkers in young and old healthy donor serum samples. For older HDs, the mean age was 58.7 ± 5.6 years (SD); for younger HDs, the mean age was 24.8 ± 2.1 years (SD). There was not a statistically significant difference in fluorescence signal for both cohorts (p > 0.05). **A)** Characteristic fluorescence images of HER2, CD24, miR21. **B)** Total fluorescence intensity quantification of HER2, CD24, miR21.


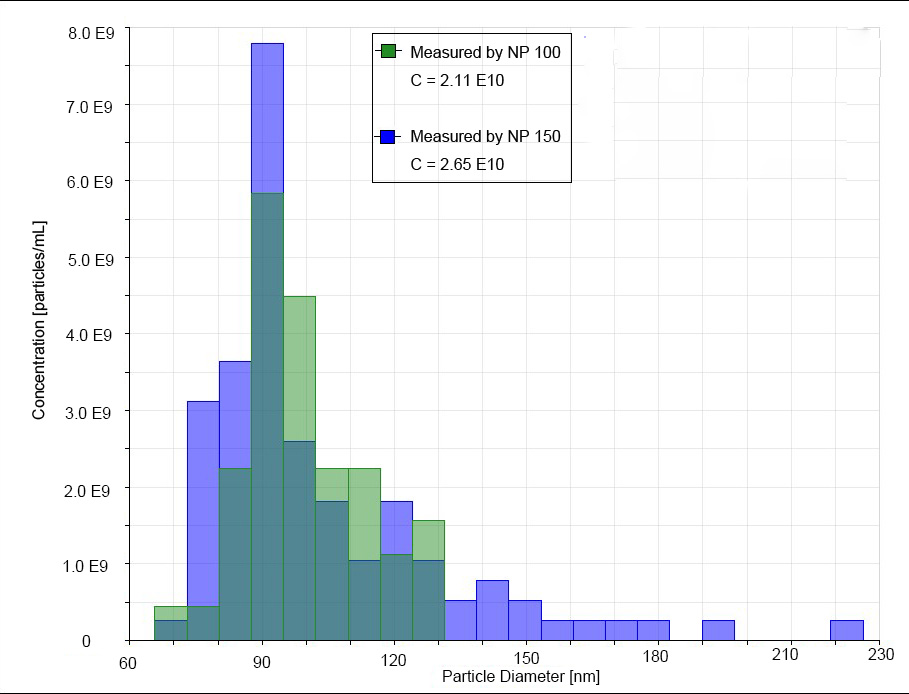


**Supplementary Figure 14.** Size distribution of EVs in a CCM sample processed by iSUF and measured by a TRPS method (qNano) using an NP100 and NP150 stretchable membrane.

**
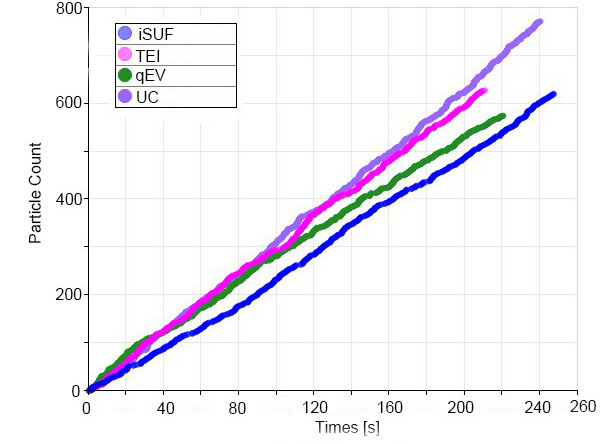
**

**Supplementary Figure 15.** Particle count plot of EVs isolated by iSUF, TEI, qEV, and UC. The graph indicates an overall constant particle detection.

**Supplementary Table 1. Comparison of 300 and 500 kDa TFF filter performance on CCM, urine, and serum.**

| **TFF filter MWCO** | **CCM (50 mL)** | | **Urine (100 mL)** | | **Serum (0.5 mL)** | |
| --- | --- | --- | --- | --- | --- | --- |
|  | **Protein removal efficiency** | **Processing time** | **Protein removal efficiency** | **Processing time** | **Protein removal efficiency** | **Processing time** |
| **300 kDa** | **>78%** | **150 min** | **>80%** | **200 min** | **>75%** | **300 min** |
| **500 kDa** | **up to 99%** | **80 min** | **up to 99%** | **100 min** | **up to 99%** | **120 min­­­­­­­-** |

**Supplementary Table 2. Protein concentration of the TFF enrichment step.**

|  | **CCM** | **Urine** | **Serum** |
| --- | --- | --- | --- |
| **Initial protein concentration (mg/mL)** | **1.20 ± 0.13 (in 50 mL)** | **1.40 ± 0.50 (in 10~100 mL)** | **10.00 ± 2.00 (dilute 0.5 mL of serum to total volume of 7mL in PBS)** |
| **Protein concentration after TFF enrichment step (mg/mL)** | **2.30 ± 0.38** | **1.90 ± 0.31** | **Not applied** |

**Supplementary Table 3. EV concentration, purity, and RNA content comparison among different EV purification platforms.**

|  | **Concentration (particles/mL)** | **Free protein (μg/mL)** | **Purity (particles/μg)** | **RNA (ng)** |
| --- | --- | --- | --- | --- |
| **qEV** | **8.2E10±3.0E10** | **15.4±4.1** | **5.9E09±3.1E09** | **8.3±7.6** |
| **UC** | **7.5E10±2.3E10** | **11841.0±2375.6** | **6.8E06±3.3E06** | **5.5±5.0** |
| **TEI** | **5.7E11±2.8E11** | **67.2±29.8** | **1.3E10±1.4E10** | **15.2±8.2** |
| **iSUF** | **4.2E12±4.7E11** | **6.3±4.3** | **1.3E12±1.3E12** | **53.7±41.0** |

**Supplementary Table 4.**

**Breast cancer patient information**

|  | **P1** | **P2** | **P3** | **P4** | **P5** |
| --- | --- | --- | --- | --- | --- |
| **Concentration**  **(NP150 sEVs/mL)** | **1.1E11** | **9.7E11** | **1.8E10** | **4.8E10** | **2.5E11** |
| **Mean size (nm)** | **124** | **130** | **127** | **139** | **120** |
| **Concentration**  **(NP300, m/l-EVs/mL)** | **9.9E9** | **1.0E11** | **9.4E9** | **1.2E10** | **9.1E10** |
| **Mean size (nm)** | **321** | **354** | **320** | **317** | **340** |
| **Age** | **66** | **55** | **81** | **60** | **78** |
| **Sex** | **F** | **F** | **F** | **F** | **F** |
| **Histology** | **Ductal carcinoma in site** | **Infiltrating duct carcinoma, NOS** | **Lobular carcinoma, NOS** | **Lobular carcinoma, NOS** | **Infiltrating duct carcinoma, NOS** |
|  | **P6** | **P7** | **P8** | **P9** | **P10** |
| **Concentration**  **(NP150 sEVs/mL)** | **5.8E10** | **1.3E11** | **3.2E10** | **9.1E10** | **3.0E9** |
| **Mean size (nm)** | **135** | **135** | **140** | **120** | **128** |
| **Concentration**  **(NP300, m/l-EVs/mL)** | **4.3E9** | **1.0E10** | **2.5E9** | **1.2E10** | **3.2E9** |
| **Mean size (nm)** | **331** | **334** | **348** | **350** | **317** |
| **Age** | **63** | **68** | **32** | **62** | **50** |
| **Sex** | **F** | **F** | **F** | **F** | **F** |
| **Histology** | **Carcinoma** | **Carcinoma** | **Infiltrating duct carcinoma, NOS** | **Adenocarci-noma** | **Adenocarci-noma** |

**Healthy donor information**

|  | **H1** | **H2** | **H3** | **H4** | **H5** |
| --- | --- | --- | --- | --- | --- |
| **Concentration**  **(NP150 sEVs/mL)** | **3.6E12** | **1.6E12** | **7.9E11** | **4.7E11** | **1.3E12** |
| **Mean size**  **(nm)** | **128** | **134** | **145** | **119** | **110** |
| **Concentration**  **(NP300, m/l-EVs/mL)** | **8.9E10** | **1.2E10** | **8.9E9** | **2.4E10** | **8.8E10** |
| **Mean size**  **(nm)** | **354** | **327** | **378** | **327** | **301** |
| **Age** | **25** | **24** | **21** | **24** | **27** |
| **Sex** | **F** | **F** | **F** | **F** | **F** |
|  | **H6** | **H7** | **H8** | **H9** | **H10** |
| **Concentration**  **(NP150 sEVs/mL)** | **2.5E12** | **3.9E11** | **4.7E12** | **2.4E11** | **9.8E10** |
| **Mean size**  **(nm)** | **128** | **134** | **119** | **122** | **107** |
| **Concentration**  **(NP300, m/l-EVs/mL)** | **2.9E11** | **1.5E11** | **1.0E11** | **1.6E10** | **1.1E10** |
| **Mean size**  **(nm)** | **324** | **354** | **320** | **317** | **305** |
| **Age** | **28** | **24** | **25** | **53** | **53** |
| **Sex** | **F** | **F** | **F** | **F** | **F** |
|  | **H11** | **H12** | **H13** | **H14** |  |
| **Concentration**  **(NP150 sEVs/mL)** | **2.7E10** | **3.2E10** | **4.2E10** | **1.1E10** |  |
| **Mean size**  **(nm)** | **124** | **120** | **134** | **127** |  |
| **Concentration**  **(NP300, m/l-EVs/mL)** | **8.5E9** | **3.2E9** | **2.9E9** | **9.5E8** |  |
| **Mean size**  **(nm)** | **328** | **317** | **322** | **300** |  |
| **Age** | **58** | **59** | **61** | **68** |  |
| **Sex** | **F** | **F** | **F** | **F** |  |
